# Supplementary material for: Renal Function Parameters in Distinctive Molecular Subtypes of Prostate Cancer
Source: Cancers (Basel). 2023 Oct 16;15(20):5013. doi: 10.3390/cancers15205013 (PMC10605320; doi:10.3390/cancers15205013)
Supplement: Supplementary file 1 [file cancers-15-05013-s001.zip › cancers-2619306-supplementary.pdf]

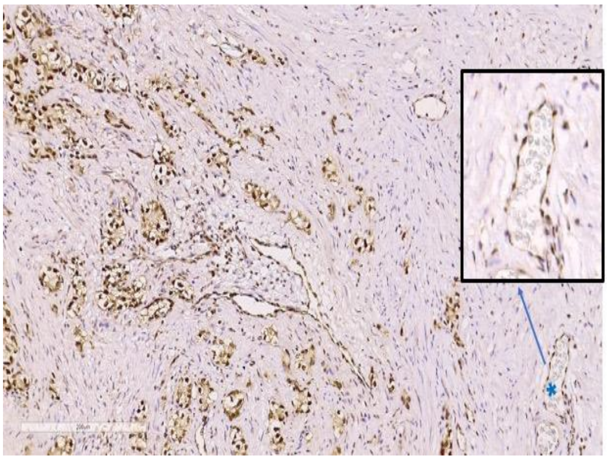

A.

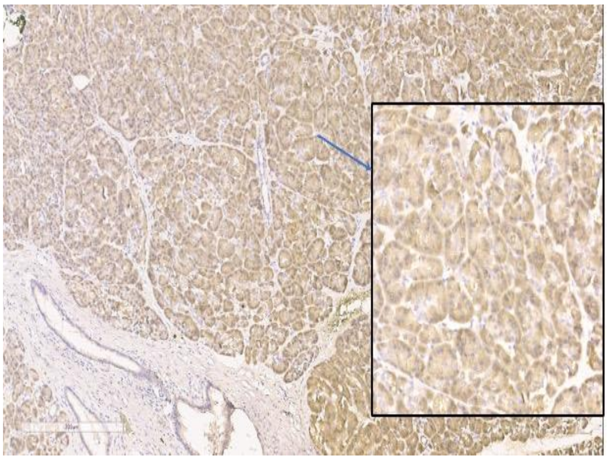

B.

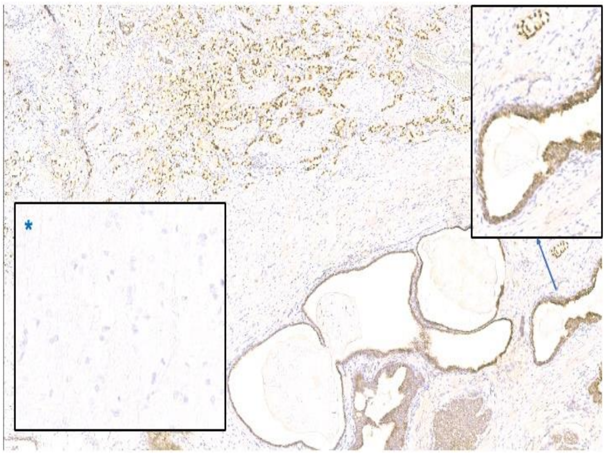

C.

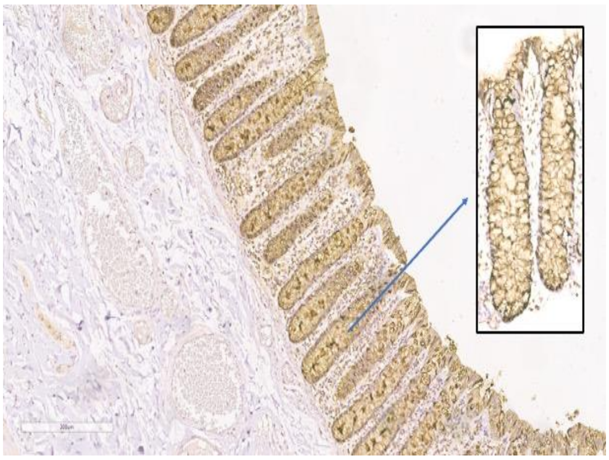

D.

Figure S1. (A–D) Positive control for the primary antibodies: **A.** anti-ERG antibody immunostaining, internal positive control nuclear expression in endothelial cells (*), H&E counterstaining, x10; **B.** anti-SPINK1 antibody immunostaining, cytoplasmic expression in acinar cells in normal exocrine pancreatic tissue, H&E counterstaining, x10; **C.** anti-HOXB13 antibody immunostaining, nuclear expression in epithelial cells of benign prostate glands (note the higher intensity in malignant cells of adjacent prostate ADK – bottom of the image) and negative control in brain tissue (*), H&E counterstaining, x10; **D.** anti- TFF3 antibody immunostaining, cytoplasmic expression in goblet cells of the colon, H&E counterstaining, x10 [29–32].
